# Pathological tooth migration-spontaneous correction of diastema after surgical periodontal therapy: a case report

**DOI:** 10.11604/pamj.2022.41.39.29953

**Published:** 2022-01-14

**Authors:** Meetu Preet Jain, Preet Rajendra Jain, Harneet Singh Chawla, Rahul Narayan Gaikwad, Om Chandrakant Wadhokar, Chaitanya Ajay Kulkarni, Waqar Mohsin Naqvi

**Affiliations:** 1 Department of Periodontics, R.R. Dental College and Hospital Umarda, Udaipur, India,; 2 Department of Prosthodontics, R.R. Dental College and Hospital Umrada Udaipur, India,; 3 Department of Oral Medicine and Radiology, PGIDS, Rohtak, Haryana, India,; 4 Department of Community Dentistry and Oral Epidemiology, College of Dentistry, Qassim University, BuraydahQassim, Kingdom of Saudi Arabia,; 5 Department of Physiotherapy, Ravi Nair Physiotherapy College, Datta Meghe Institute of Medical Sciences, Sawangi(M), Wardha, India,; 6 Department of Community Health Physiotherapy, Ravi Nair Physiotherapy College, Datta Meghe Institute of Medical Sciences, Sawangi (M), Wardha, India,; 7 Adjunct Faculty, MGM School of Physiotherapy Aurangabad, MGM Institute of Health Sciences, Navi Mumbai, Maharashtra, India

**Keywords:** Diastema, periodontitis, pathologic tooth migration, splinting, case report

## Abstract

A common consequence of moderate to extreme periodontitis is pathologic migration. This denotes tooth movement when the periodontal disease interjects the equilibrium among the elements that preserve physiological tooth position. The balancing factors can migrate the teeth in any direction. The etiology of pathological migration tends to be multifactorial, thus achieving early diagnosis is imperative, which will ultimately lead to the prompt removal of the etiological factors while avoiding severe bone destruction. In this case maxillary central incisors had diastema due to pathological migration with mobility grade I in maxillary left central incisor. Many cases of moderate to severe pathological migration need a suitable, interdisciplinary approach. Nevertheless, since it is possible to detect mild cases of Pathological tooth migration (PTM) at an early stage, spontaneous correction of migrated teeth can be accomplished by periodontal therapy alone. Regardless of the treatment selected, maintenance of stable results should be considered as an aim of treatment.

## Introduction

Pathological of anterior-teeth is a common trigger of esthetic distress seen among adults. Pathological migration is characterized as a shift in the location of the tooth resulting from disturbance of the forces that hold the teeth in normal position in relation to their arch [1]. The prevalence of PTM among periodontal disease patients varies from 30.03% to 55.8% [2-4]. This can be caused by many etiological factors, including periodontal loss of attachment, inflamed tissue pressure, occlusal factors, habits such as tongue thrusting and bruxism, non-rehabilitation of lost teeth, gingival enlargement, and iatrogenic factors. Anterior pathological migration of the teeth poses both functional and esthetic problems [5-7]. The purpose of this article is to present a case following periodontal therapy with spontaneous closure of the diastema [8].

## Patient and observation

**Patient information:** a 38-year-old woman without any systemic history reported to the Hospital´s Dentistry Department with a chief complaint of dull pain in the upper front jaw area.

**Figure 1:**
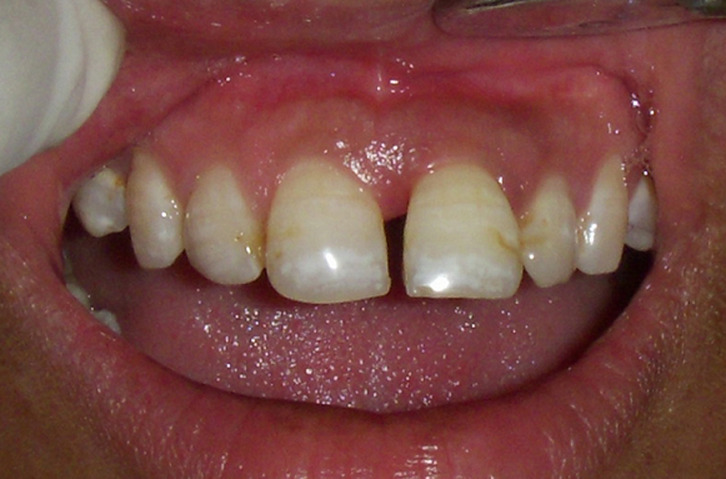
pre-operative midline diastema

**Clinical findings:** while there were no local factors present at the initial examination and the patient had good oral hygiene, the upper left central incisor (UL1) showed distolabial migration with extrusion, thereby producing midline diastema of 2.5mm (Figure 1). An UNC-15 periodontal probe was used around the tooth and revealed a deep pocket of 8mm with grade I mobility.

**Figure 2:**
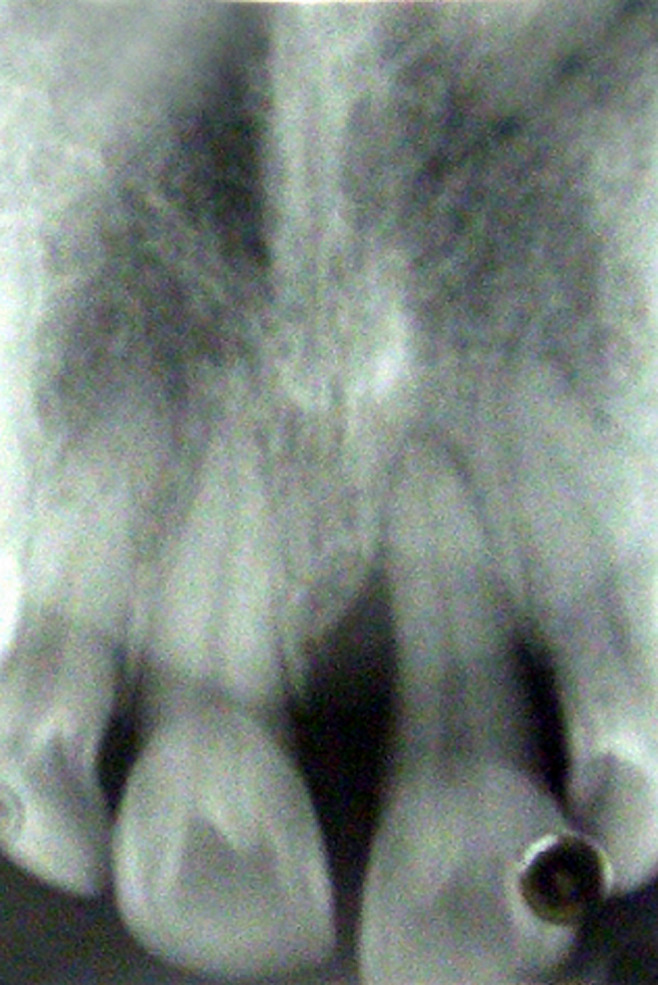
X-ray shows periapical radiolucency

**Diagnostic findings:** radiographic observation showed severe vertical bone loss, periodontal ligament space widening with periapical radiolucency (Figure 2). Tooth was found to be non-vital after pulp testing. Diagnosis of chronic localized advanced periodontitis was made.

**Figure 3:**
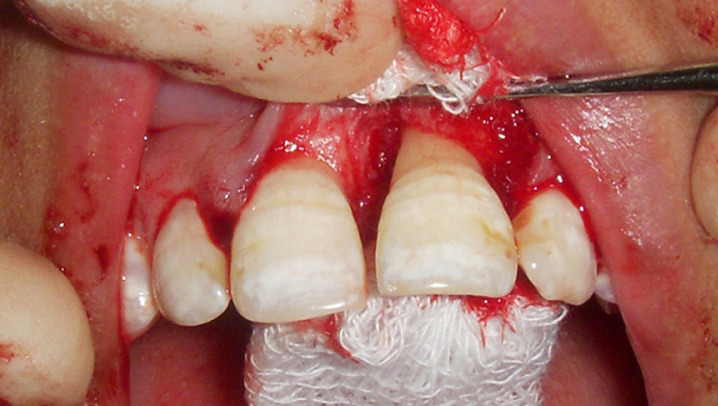
post debridement

**Figure 4:**
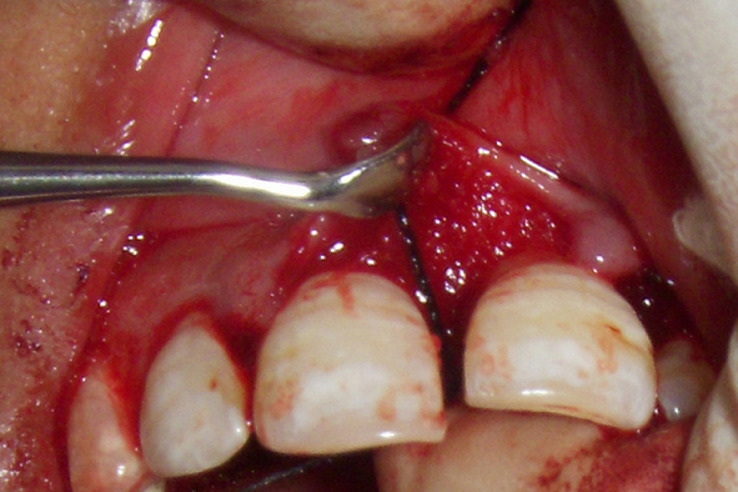
defect treated with bone graft

**Figure 5:**
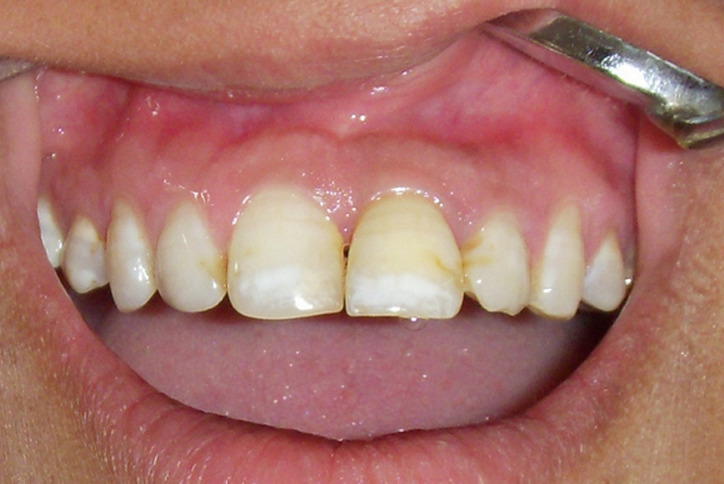
midline diastema closed completely

**Therapeutic intervention:** the treatment plan for this patient was endodontic intervention for upper left central incisor (UL1) followed by elimination of periodontal inflammation by regenerative periodontal surgery. After subgingival scaling and root planning, oral hygiene instructions were given. Occlusal interferences were checked, and occlusal adjustment was performed to prevent trauma from occlusion. One month later, although the clinical signs of gingival inflammation had disappeared, a deep pocket was left in relation to the treated tooth. According to the treatment plan, regenerative periodontal therapy using GeistlichBio-Oss® was performed. Following local anesthesia, horizontal and vertical releasing incisions were performed. A full-thickness flap was elevated. The defect was completely debrided, and the surfaces were carefully scaled and planed (Figure 3). The defect was treated with bone graft material (Figure 4). The flap was then sutured and packed with Coe-Pak™. Post-surgical standard antibiotics' regimen was prescribed to the patient. The suture removal was done after one week of the surgery. The patient was kept on maintenance therapy at 1-2 months´ interval for six months. It was observed that after this stipulated time of six months, diastema was completely closed (Figure 5). At this appointment, full-mouth professional prophylaxis and a fiber reinforced splint was given to maintain the position of the tooth.

**Timeline:** the individual was diagnosed and randomized controlled trial (RCT) and flap surgery was done on February 15^th^, 2020, suture were removed on January 22^th^, 2021, the individual was given splinting on January 1^st^, 2021.

**Patient perspective:** due the pain I was not able to concentrate on my work and having meals and chewing was difficulty after undergoing surgery the pain was subsided. The surgery along with medications help me a lot in reducing the pain and going back to normal.

**Informed consent:** the patient was explained about the report and an oral consent was obtained from the patient.

## Discussion

Periodontal bone loss is one of the main contributing factors of PTM. Martinez-Canut *et al*. studied periodontal patients, and found bone deterioration associated with PTM. When bone loss increased, PTM's likelihood rose from 2.95 to 7.97 times. In another set of periodontal patients, Towfighi *et al*. measured the loss of attachment on migrated and non-migrated contralateral teeth and found that the loss of attachment of migrated teeth was substantially greater than that of non-migrated teeth. Costa and associates observed a few forms of PTM, including extrusion and facial flaring, and it was important to note that the teeth being extruded and flaring, respectively, displayed around 59% and 45% bone loss. A multitude of options have been suggested for the management of specific ranges of PTM. There have been several literature schools of thought concerning the treatment of severe PTM which often include a multidisciplinary approach where periodontal and adjunctive orthodontic and/or prosthodontic intervention may be needed, while some relevant previous case studies suggested that periodontal therapy may trigger spontaneous correction of the PTM in some patients [9,10].

The authors of this study concluded that if a newly developed anterior teeth diastema associated with periodontal disease is ≤1 mm in size, closure after periodontal therapy is predictable [6]. Nevertheless, some research suggested closure of even larger spaces. Sato *et al*. reported a case in which nonsurgical periodontal treatment resulted in spontaneous correction of pathologic tooth migration, including 3mm diastema [11]. This is in accordance with the finding of our report. In the current context, the tooth (Maxillary left central incisor) had proclined labially and incisally, causing diastema. Chronic periodontitis has contributed to bone loss around the tooth of 30 to 50 per cent. Based on the clinical radiographic analysis, we felt the bone loss was the key explanation for PTM occurrence [12]. The causes of these corrections can be explained as follows: removal of the detrimental effects of bacterial infection resulting in a reduction in inflammatory tissue strain, restoration of the “periodontal force” by healing the supracrestal gingival tissues, especially transseptal fibers [13], and regeneration of collagen in gingival fiber apparatus due to the eradication of traumatic occlusal forces [6]. Since we cannot restore the missing bone, and particularly during function, we are incapable to escape the impact of trauma from occlusion and soft tissue, further tooth migration can occur. We had therefore decided to secure the tooth by placing a splint in position [6].

## Conclusion

Several patient attributes affect pathologic tooth migration’s treatment choice. Periodontal disease control can deliver the single most effective approach of preventing PTM. Effective treatment of pathological tooth migration (PTM) includes early diagnosis of its etiopathogenesis and intervention associated with the particular etiology. As most cases of moderate to serious PTM also involve an interdisciplinary approach to success. However early diagnosis of PTM, spontaneous correction of migrated teeth can be accomplished by periodontal therapy alone. Regardless of the treatment chosen, inherent stability of the tooth position should be deliberated as an objective of treatment modality.
